# The Effect of Sperm DNA Fragmentation on *In Vitro* Fertilization Outcomes for Women With Polycystic Ovary Syndrome

**DOI:** 10.3389/fendo.2022.822786

**Published:** 2022-05-27

**Authors:** Huan Wang, Hui Li, Jing Zhu, Jianmin Xu, Yuqing Jiang, Wenhui Chen, Yingpu Sun, Qingling Yang

**Affiliations:** ^1^ Center for Reproductive Medicine, The First Affiliated Hospital of Zhengzhou University, Zhengzhou, China; ^2^ Henan Key Laboratory of Reproduction and Genetics, The First Affiliated Hospital of Zhengzhou University, Zhengzhou, China; ^3^ Henan Provincial Obstetrical and Gynecological Diseases (Reproductive Medicine) Clinical Research Center, The First Affiliated Hospital of Zhengzhou University, Zhengzhou, China

**Keywords:** PCOS, sperm DNA fragmentation index (DFI), *in vitro* fertilization, embryonic development, pregnancy outcome

## Abstract

**Background:**

Polycystic ovary syndrome (PCOS) is a prevalent endocrine disease in reproductive women associated with poor pregnancy outcomes. In modern society, people pay more attention to the female factor, but it is uncertain whether sperm quality is another factor affecting pregnancy outcomes of patients with PCOS.

**Method:**

The effect of sperm DNA fragmentation (SDF) on oocyte fertilization, embryonic development, and pregnancy outcomes for patients with PCOS who underwent *in vitro* fertilization (IVF) treatment was studied. A total of 141 PCOS patients and 332 control patients undergoing IVF treatment were recruited from January 2017 to December 2019. All female patients were designated into two groups according to the Rotterdam criteria. Each group was divided into two sets, DNA fragmentation index (DFI) ≤15% and DFI > 15%, on the basis of sperm DFI.

**Result:**

There were no differences in basic clinical characteristics between couples with a sperm DFI ≤ 15% or DFI > 15%. For control patients, no differences were observed in IVF outcomes. However, for PCOS patients, although there were no differences in the fertilization (60.4% vs. 59.9%, p = 0.831), high-quality embryo (68.5% vs. 67.9% p = 0.832), clinical pregnancy (78.4% vs. 66.7%, p = 0.148), and abortion (12.5% vs. 11.5%, p = 1.000) rates, a significantly lower high-quality blastocyst formation rate (26.3% vs. 16.3%, p = 0.023) was observed for couples with a sperm DFI > 15% compared with a sperm DFI ≤ 15%.

**Conclusion:**

For PCOS patients undergoing IVF, oocytes fertilized using sperm with higher DFI led to a lower high-quality blastocyst formation rate but had no influence on fertilization, high-quality embryo, clinical pregnancy, and miscarriage rates.

## Introduction

Polycystic ovary syndrome (PCOS) is one of the most widespread gynecological endocrine conditions in women of childbearing age and is generally considered to be an important public health problem (1, 2). In particular, PCOS is accompanied by infertility, menstrual absence or irregularity, hyperandrogenism and/or hyperandrogenemia, and obesity (3) and affects approximately 6%–15% of adult women (4, 5). During human ovarian follicular development, many primordial follicles are recruited as a group of growing follicles, and then a dominant follicle will ovulate. However, in patients with PCOS, ovarian hyperandrogenism and regulatory systems disrupt follicular development, which results in ovulation dysfunction (2, 6). Although increasing evidence shows that patients with PCOS produce more oocytes during treatment *via* assisted reproductive technology, clinical pregnancy outcomes remain disappointing (7–9). Studies have demonstrated in patients with PCOS that there are a lower fertilization rate and higher risk of early spontaneous miscarriage (10–12), associated with poor oocyte and embryo quality (13–16). A meta-analysis showed that women with PCOS had a significantly elevated risk of miscarriage and premature delivery (17) and that their babies had a significantly elevated risk to be treated at a neonatal intensive care unit (OR: 2.31; 95% CI: 1.25–4.26) and higher postpartum mortality (OR: 3.07; 95% CI: 1.03–9.21) (18).

The integrity of sperm DNA plays a major role in fertilization and the development of healthy offspring (19), so the sperm DNA fragmentation index (DFI) may be an important clinical indicator for figuring out the extent of sperm DNA damage and integrity (20). There is clear evidence that infertile men have a higher amount of sperm DFI than fertile men (21). Other studies pointed out that high sperm DFI was connected with decreased fertilization, high-quality embryo development, and conception rates and increased risk of abortion (22–25), as well as the risk of genetic diseases in offspring (21).

The genomic integrity of a zygote is indispensable for normal embryo development. Human sperm has a limited ability to repair DNA damage, so repairing sperm DNA damage is dependent on oocytes (26). Although an oocyte can repair DNA damage in a fertilizing spermatozoon, the capacity is limited. For example, studies showed that pregnancy outcomes were associated with the degree of DNA damage (27) and that oocytes could repair sperm DNA damage when it was less than 8% (28). For patients with PCOS, it remains uncertain whether oocytes have a disrupted ability to repair sperm DNA fragmentation (SDF). Therefore, this research observed whether the different extent of sperm DNA damage had an influence on embryonic development and clinical pregnancy outcomes for patients with PCOS undergoing *in vitro* fertilization (IVF) treatment.

## Material and Methods

### Patients and Experimental Design

This study enrolled a total of 473 infertile couples who underwent IVF for the first time at the Reproductive Medical Center of the First Affiliated Hospital of Zhengzhou University from January 2017 to December 2019. All couples had normal chromosome karyotypes and had cleavage-stage embryos or blastocysts for transfer. Male age was less than 45 years, and all male partners had recently not taken any drug that was harmful to the sperm. According to the Rotterdam criteria, all patients were divided into two groups, PCOS (n = 141) and control (n = 332), and each group was divided into two sets on the basis of sperm DFI: DFI ≤ 15% and DFI > 15%. The inclusion criteria of control patients in the study were women aged less than 35 years with a basal level of follicle-stimulating hormone (FSH) < 10 mIU/ml, 18.5 < body mass index (BMI) ≤ 24.9, and female infertility only due to fallopian tube dysfunction. Based on the 2003 Rotterdam criteria (29), PCOS patients should meet at least two of the three following characteristics: 1) oligo/anovulation, 2) clinical symptoms caused by high testosterone and/or hyperandrogenemia, and 3) ultrasonography showing more than 12 follicles with diameters of 2–9 mm in one or both ovaries and/or the volume of one ovarian >10 ml. The inclusion criteria of PCOS patients were as follows: 1) women were less than 35 years old, 2) female infertility was due to fallopian tube factor, and 3) a basal level of FSH < 10 mIU/ml.

### Semen Analysis

All examinations were evaluated on the basis of the WHO laboratory manual (30). Semen samples were collected into sterile and non-poisonous containers by masturbation after 3 to 7 days of abstinence, and the samples were kept in a controlled environment to avoid changes that will affect sperm quality. After liquefaction for 30 min, sperm concentration and viability were examined in a Makler chamber (Sefi Medical Instruments, Haifa, Israel). In accordance with Kruger’s strict criteria, sperm morphology assessment was detected using H&E staining. After liquefaction, semen samples were prepared using two discontinuous density gradients (80%–40%), and the precipitate was collected after centrifugation. Then the samples were washed twice with IVF medium, and the final precipitant was suspended in a suitable volume of IVF medium.

### Sperm DNA Fragmentation Index Detection

Semen was collected after 3 to 7 days of abstinence, and the sperm DFI was tested using sperm chromatin structure assay (SCSA) after liquefaction. The semen density was adjusted to (0.5–1.0) × 10^6^/ml with TNT buffer, and the semen sample was incubated with acridine orange (AO; PH 6.0) solution. Then the sperm were analyzed by a flow cytometer (BD FACS Canto II, BD Biosciences, San Jose, CA, USA) and calculated by specific software to analyze the fluorescence signals. The double-stranded DNA emitted green fluorescence when binding to AO, while single-stranded DNA emitted red fluorescence. The number of sperm was not less than 5,000, and the DFI was calculated by measuring the percentage of sperm with red fluorescence in the total sperm number. At present, there are three major methodologies to test SDF, namely, the comet assay, the terminal deoxynucleotidyl transferase-mediated fluorescein-dUTP nick end labeling (TUNEL) assay, and the SCSA. In comet assay, only 50–200 sperm cells per sample are measured by a fluorescence microscopic test, which lacks statistical robustness. Although the TUNEL assay can be done by flow cytometry, it also can be applied in bright field/fluorescence microscopy, which has much less statistical robustness. However, the SCSA is different from other tests, which is carried out under a normalized protocol (31) and has definite and clinically valuable cutoff levels for evaluating spermatogenic potential (32). In SCSA, 5,000–10,000 sperm cells can be analyzed within a few seconds, which can provide precise and repeatable statistical data. Although the variation of SCSA analysis among laboratories is relatively stable, the implementation rate of SCSA is lower because of an expensive flow cytometer.

### Controlled Ovarian Stimulation and Laboratory Procedures

Patients undergoing IVF used an ultra-long protocol for promoting ovulation. On the second or third day of the menstrual cycle, the patients were injected intramuscularly with a gonadotropin-releasing hormone agonist (GnRH-a; triptorelin acetate) to reduce gonadotropin secretion in the pituitary gland. After 28–35 days, patients were assessed by color Doppler ultrasound inspection and routine blood examinations for FSH, luteinizing hormone, and estradiol levels. When the gonadotropin levels were at a low threshold, induction of ovulation was implemented, and the development of follicles was monitored by transvaginal ultrasound inspection. When the number of the mean diameter of dominant follicles with at least 17 mm was more than three, the usage of gonadotropin was ceased. Then the patients were injected intramuscularly with 10,000 IU of recombinant human chorionic gonadotropin (HCG, Livzon) to promote final follicle maturity. Oocytes were collected 35–37 h after injection.

### Oocyte Fertilization and Embryo Culture

Fresh sperm were prepared using density gradient centrifugation in sperm-gradient 40% and 80% solutions, and the collected sperm underwent sperm swim-up technique to reach a final sperm concentration of 1 × 10^6^/ml. Then sperm were cultured with mature oocytes for fertilization. Embryos were cultured in a comfortable atmosphere of 6% CO_2_ at 37°C. Then the physician decided to use two good-quality grade I or II embryos (using the Peter scoring system) on Day 3 or one blastocyst for transfer per patient based on embryo quality and the physical condition of the patients. The rate of fertilization was calculated by measuring the proportion of double pronuclear embryos (2PN) in total MII oocytes. The rate of high-quality embryos was defined as the number of embryos with grade I or II/the number of 2PN cleavage. The blastocysts were evaluated according to Gardner and Schoolcraft scoring system, and high-quality blastocysts were defined as those scoring ≥3 BB. The rate of high-quality blastocyst formation was defined as the number of high-quality blastocysts/total developed blastocysts.

### Clinical Follow-Up

When the embryo was transferred at 14 days, the patients were tested for serum β-hCG levels. Serum β-hCG levels >50 IU/L were diagnosed as biochemical pregnancy. When the embryo transferred at 35 days, the patients were asked to get a transvaginal ultrasound. Detecting a normal fetal heart rate in the uterus was considered a clinical pregnancy. Abortion was defined as an embryo or fetus that did not survive.

### Statistical Analysis

Statistical data were analyzed by SPSS 25.0 software. Variable data were shown as the mean ± SD (x ± s). The rates of early embryo development, clinical pregnancy, and abortion were contrasted using the χ^2^ test. p < 0.05 was defined as statistical significance.

## Results

### Relationships Between Sperm DNA Fragmentation and *In Vitro* Fertilization Outcomes for Women With Control and Polycystic Ovary Syndrome

As shown in **Table 1**, the differences in basic clinical characteristics (age, BMI, and basal levels of hormone) between the couples with a sperm DFI ≤ 15% and a sperm DFI > 15% were not observed, but embryonic development differed between the two groups; all results are shown in **Table 2**.

**Table 1:** The basic clinical characteristics in control and PCOS patients with different sperm DFI.

Variable	Control (n = 332)			PCOS (n = 141)		
	DFI ≤ 15(n = 246)	DFI > 15(n = 86)	p	DFI ≤ 15(n = 102)	DFI > 15(n = 39)	p
Female age (years)	29.5 ± 2.8	29.8 ± 2.9	0.325	28.6 ± 3.3	28.2 ± 3.5	0.444
Female BMI (kg/m^2^)	21.4 ± 1.7	21.6 ± 1.8	0.559	24.4 ± 3.4	24.1 ± 3.9	0.621
FSH (mIU/ml)	6.9 ± 1.8	7.2 ± 2.0	0.193	6.1 ± 1.5	5.9 ± 1.8	0.412
E2 (pg/ml)	41.4 ± 32.2	40.4 ± 23.1	0.786	45.3 ± 33.8	50.4 ± 43.2	0.474
P4 (ng/ml)	0.4 ± 0.3	0.5 ± 1.8	0.448	0.6 ± 1.1	0.6 ± 0.6	0.905
LH (mIU/ml)	5.7 ± 3.8	6.0 ± 6.1	0.586	9.8 ± 5.7	9.6 ± 7.6	0.922
Male age (years)	30.4 ± 4.1	31.2 ± 4.4	0.112	29.3 ± 3.6	29.4 ± 3.9	0.960
Male BMI (kg/m^2^)	25.0 ± 3.8	25.1 ± 3.5	0.967	25.0 ± 3.6	24.4 ± 3.8	0.425
Sperm concentration (%)	53.1 ± 33.5	44.0 ± 26.4	0.013	58.6 ± 36.4	44.4 ± 26.7	0.03
DFI (%)	8.5 ± 3.3	21.8 ± 7.5	0.000	8.5 ± 3.3	22.4 ± 6.5	0.000
Progressive motility	40.2 ± 7.5	34.6 ± 8.4	0.000	40.2 ± 9.1	32.2 ± 11.4	0.000

Data are presented as mean ± SE. DFI, DNA fragmentation index; BMI, body mass index; FSH, follicle-stimulating hormone; E2, estradiol; P4, progesterone; LH, luteinizing hormone.

**Table 2:** IVF-ET laboratory characteristics in control and PCOS patients with different sperm DFI.

	Control (n = 332)			PCOS (n = 141)		
	DFI ≤ 15(n = 246)	DFI > 15(n = 86)	p	DFI ≤ 15(n = 102)	DFI > 15(n = 39)	p
Retrieved oocytes (n)	14.0 ± 5.8	12.4 ± 5.4	0.034	16.2 ± 6.0	15.7 ± 4.9	0.672
Mature oocytes (n)	11.4 ± 5.0	9.9 ± 4.9	0.015	13.2 ± 4.9	12.5 ± 4.5	0.703
Fertilization rate (%)	76.2% (2125/2788)	78.8% (660/838)	0.127	76.4% (996/1304)	77.1% (367/476)	0.751
Good embryos (%)	72.7% (1520/2090)	70.0% (458/654)	0.180	68.5% (673/983)	67.9% (247/364)	0.832
Blastocyst development (%)	60.2% (742/1232)	62.8% (218/347)	0.381	57.8% (339/586)	60.0% (129/215)	0.584
High-quality blastocysts (%)	31.3% (232/742)	32.1% (70/218)	0.814	26.3% (89/339)	16.3% (21/129)	0.023

Data are presented as mean ± SE, unless otherwise noted. IVF, in vitro fertilization; PCOS, polycystic ovary syndrome; DFI, DNA fragmentation index.

We analyzed the relationship between sperm DFI and embryonic development in control and PCOS patients. Although there were no significant differences for the control couples undergoing IVF, for PCOS patients, a lower high-quality blastocyst rate (26.3% vs. 16.3%; p = 0.023) was observed for couples with a sperm DFI > 15% compared with a sperm DFI ≤ 15%. Surprisingly, no statistical differences between SDF and fertilization and high-quality embryo rates in the two groups were observed.

To further explore whether SDF had an impact on pregnancy outcomes, we analyzed the correlation between different damaged DNA and the rates of clinical pregnancy and abortion in two groups, as shown in **Table 3**. Surprisingly, no statistical differences regarding clinical pregnancy (78.4% vs. 66.7%, p = 0.148) and abortion (12.5% vs. 11.5%, p = 1.000) rates in two groups were observed.

**Table 3:** IVF-ET pregnancy outcomes in control and PCOS patients with different sperm DFI.

	Control (n = 332)			PCOS (n = 141)		
	DFI ≤ 15(n = 246)	DFI > 15(n = 86)	p	DFI ≤ 15(n = 102)	DFI > 15(n = 39)	p
Embryos transferred (n)	1.7 ± 0.5	1.7 ± 0.4	0.247	1.6 ± 0.5	1.7 ± 0.5	0.397
Implantation rate (%)	54.4% (223/410)	49.0% (73/149)	0.258	61.7% (100/162)	52.3% (34/65)	0.192
Clinical pregnancy rate (%)	69.5% (171/246)	65.1% (56/86)	0.450	78.4% (80/102)	66.7% (26/39)	0.148
Abortion rate (%)	8.2% (14/171)	5.4% (3/56)	0.685	12.5% (10/80)	11.5% (3/26)	1.000

Data are presented as mean ± SE, unless otherwise noted. IVF, in vitro fertilization; PCOS, polycystic ovary syndrome; DFI, DNA fragmentation index.

## Discussion

It is established that PCOS is a prevalent metabolic disease that negatively affects female reproduction, health, and quality of life (4). Most research has mainly focused on female infertility factors related to PCOS. However, poor pregnancy outcomes for PCOS women may be related not only to oocytes but also to sperm quality. Sperm DNA integrity plays an important role in precisely transmitting parental genetic material to the offspring (33, 34). Increasing evidence supported the view that high sperm DFI was associated with poor pregnancy outcomes, regardless of natural conception and fertility treatments (35, 36). A recent meta-analysis study pointed out that sperm DNA damage may adversely affect clinical gestation after patients underwent IVF and/or intracytoplasmic sperm injection (ICSI) treatment (37). The association between higher levels of sperm DFI and increased early abortion rate was observed in a retrospective cohort study (20), and a prospective observational study indicated that in ICSI cycles, elevated sperm DNA damage had a negative influence on pregnancy rates (38). However, few studies have explored the effect of sperm DFI on embryonic development and reproductive outcomes of patients with PCOS. Therefore, the current study investigated the effect of sperm DFI on embryonic development and fetation outcomes.

In this research, higher sperm DFI had no influence on the fertilization rates in patients with PCOS, which is consistent with previous reports that showed that sperm DNA is not involved in the fertilization process, even though spermatozoa with fragmented DNA could fuse with oocytes to begin the process of fertilization (33, 39–42). Before embryonic genes are activated, repair of paternal DNA is attributed to maternal RNA and proteins controlling DNA integrity (43, 44). Recent studies demonstrated that declining oocyte quality is positively correlated with diminished efficiency of DNA repair and negatively correlated with embryo development (23, 45). For patients with PCOS, the present study observed that oocytes fertilized with higher levels of SDF (>15%) resulted in a poor-quality blastocyst rate, which supports the work by Fatehi et al., which showed that oocytes fertilized with damaged sperm DNA exhibited normal 2–3 cleavage stage development but were blocked at the blastocyst stage (46). To a certain extent, oocytes can repair SDF, and good-quality oocytes can repair more damage (47). For example, a prospective observational study suggested that in donor cycles, an elevated sperm DFI had no influence on embryo development because of using the good-quality donated oocytes, which were sufficiently capable of repairing DNA damage (33). We speculated that oocytes from women with PCOS had a reduced ability to repair DNA damage, which led to lower high-quality blastocyst rates. Another study showed that in ICSI cycles in women of the advanced age group, the capacity of oocytes to repair sperm DNA damage was reduced, which led to lower high-quality Day 3 embryo implantation and conception rates and higher abortion rates (23). Conversely, the current findings showed no difference in clinical pregnancy and miscarriage rates for patients with PCOS using sperm with >15% DFI compared with ≤15% DFI. Our results show that elevated SDF had no effect on pregnancy outcomes in patients with PCOS because of the selection of the best embryos for transfer.

PCOS is a complex endocrine disorder, and sperm DNA damage in the current study may not be the only factor that influences embryonic development. Other factors intrinsic to patients with PCOS may have impacted negatively upon embryonic development. Also, in our study, semen was prepared using density gradient centrifugation to select optimal sperm fertilization. Therefore, one reason for no difference in clinical pregnancy and miscarriage rates between patients with PCOS using sperm with a DFI ≤ 15% and > 15% may be the preoptimized sperm. It is urgent to do more research to reveal the underlying mechanism by which increased sperm DNA damage is associated with reduced embryonic development for women with PCOS who undergo fertility treatments.

Although traditional semen analysis is important to evaluate male infertility (48), it does not fully reflect the fertility potential of sperm. Numerous studies have shown that about 15% of patients with normal semen parameters exhibit infertility (48, 49). Therefore, it is necessary to identify diagnostic measures to detect subtle sperm defects, such as chromosomal condensation defects or DNA strand breaks.

The study was limited by the number of patients in the PCOS group because a small sample size was more likely to produce research bias. Otherwise, as a retrospective study, there was no chance to detect the sperm DFI of the treated sperm sample. To further study whether higher sperm DFI can influence IVF outcomes of PCOS women, the sample size should be expanded, and it is better to evaluate the sperm DFI of treated sperm samples to exclude the influence during the pretreatment process in IVF cycles.

In conclusion, in the two groups, there was no difference between fertilization, high-quality embryo rates, and higher DFI (DFI > 15%). However, there was a significant statistical difference between the rate of good-quality blastocysts and higher DFI (26.3% vs. 16.3%, p = 0.023) for patients with PCOS undergoing IVF treatment. Surprisingly, clinical pregnancy and miscarriage rates were independent of SDF, because of the selection of the best embryos or blastocyst for transfer.

## Data Availability Statement

The original contributions presented in the study are included in the article/supplementary material. Further inquiries can be directed to the corresponding authors.

## Ethics Statement

The studies involving human participants were reviewed and approved by the First Affiliated Hospital of Zhengzhou University. The patients/participants provided their written informed consent to participate in this study.

## Author Contributions

YS and QY made contributions to the study conception and design. QY and HW were responsible for the article drafting, data analysis, and interpretation. HW and HL took part in the result discussion. JZ, JX, YJ, and WC participated in data collection. All authors listed have made a substantial, direct, and intellectual contribution to the work and approved it for publication.

## Funding

The study was supported by the National Key R&D Program of China 2019YFA0110900 and Key Program for International Science and Technology Cooperation Projects of China 81820108016 to YS and the National Natural Science Foundation of China 31970800 to QY.

## Conflict of Interest

The authors declare that the research was conducted in the absence of any commercial or financial relationships that could be construed as a potential conflict of interest.

## Publisher’s Note

All claims expressed in this article are solely those of the authors and do not necessarily represent those of their affiliated organizations, or those of the publisher, the editors and the reviewers. Any product that may be evaluated in this article, or claim that may be made by its manufacturer, is not guaranteed or endorsed by the publisher.
